# A systematic review of the literature on the effectiveness of exercise therapy for groin pain in athletes

**DOI:** 10.1186/1758-2555-1-5

**Published:** 2009-03-31

**Authors:** Zuzana Machotka, Saravana Kumar, Luke G Perraton

**Affiliations:** 1 Centre for Allied Health Evidence, University of South Australia, North Terrace, Adelaide, South Australia, 5000, Australia

## Abstract

**Background:**

Athletes competing in sports that require running, changes in direction, repetitive kicking and physical contact are at a relatively higher risk of experiencing episodes of athletic groin pain. To date, there has been no systematic review that aims to inform clinicians about the best available evidence on features of exercise interventions for groin pain in athletes. The primary aim of this systematic review was to evaluate the available evidence on the effectiveness of exercise therapy for groin pain in athletes. The secondary aim of this review was to identify the key features of exercise interventions used in the management of groin pain in an athletic population.

**Methods:**

MEDLINE, CINAHL, PubMed, SPORTSDiscus, Embase, AMED, Ovid, PEDro, Cochrane Controlled Trials Register and Google Scholar databases were electronically searched. Data relating to research design, sample population, type of sport and exercise intervention was extracted. The methodological evaluation of included studies was conducted by using a modified quantitative critical appraisal tool.

**Results:**

The search strategy identified 468 studies, 12 of which were potentially relevant. Ultimately five studies were included in this review. Overall the quality of primary research literature was moderate, with only one randomised controlled trial identified. All included studies provided evidence that an exercise intervention may lead to favourable outcomes in terms of return to sport. Four of the five studies reviewed included a strengthening component and most utilised functional, standing positions similar to those required by their sport. No study appropriately reported the intensity of their exercise interventions. Duration of intervention ranged from 3.8 weeks to 16 weeks. All five studies reported the use of one or more co-intervention.

**Conclusion:**

Best available evidence to date, with its limitations, continues to support common clinical practice of exercise therapy as a key component of rehabilitation for groin pain in athletes. Overall, the available evidence suggests that exercise, particularly strengthening exercise of the hip and abdominal musculature could be an effective intervention for athletes with groin pain. Literature provides foundational evidence that this may need to be in the form of progressive exercises (static to functional) and performed through range. There is currently no clear evidence regarding the most effective intensity and frequency of exercise, because of a lack of reporting in the primary literature.

## Background

Groin pain associated with and aggravated by sporting activity is a common complaint in the athletic population. Resulting pain can be local or diffuse and can arise from one or more musculoskeletal sources including the lumbar spine, hip joint, anterior pelvis, adductor musculo-tendinous unit and lower abdominal wall [1]. Pain is considered a consequence of acute and/or long-standing injury and may include features consistent with a chronic pain state [2]. There are also a number of non-musculoskeletal presentations such as infection, tumour, gynaecological and digestive conditions that are not associated with sporting activity and should be investigated early if suspected as a potential source of pain [3].

Athletes competing in sports that require running, especially changes in direction whilst running, repetitive kicking and physical contact are at highest risk of experiencing episodes of athletic groin pain [3,4]. Documented high risk sports include Australian football, soccer, rugby and ice hockey [5-8]. A recent systematic review by Maffey and colleagues [9] investigated risk factors and injury prevention strategies for groin strain injury in sport. One of the important findings of this review was the lack of uniform consensus on the definition of groin strain injury. This review also identified a distinct lack of differentiation between acute and chronic groin presentations and their associated signs and symptoms.

To date, there is evidence highlighting the complexity in the presentation and diagnosis of groin pain in athletes [10,11]. Ekberg and colleges [10] found 19 out of 21 athletes in their sample of longstanding groin pain, which excluded hernia presentations, tested positive for two or more likely pathologies for the source of their groin pain. Lovell [11] found 27% of 189 athletes with chronic groin pain were found to have multiple pathologies with 50% testing positive for incipient hernia. Hence diagnosis of the source of groin pain in athletes is often difficult.

A recent systematic review by Jansen and colleagues [12] which reviewed diagnostic procedures for athletes presenting with long standing groin pain concluded that following clinical examination, Magnetic Resonance Imaging (MRI) and ultrasound should be utilised as the primary diagnostic tools. However, appropriate clinical examination may not always occur and diagnostic equipment may not always be readily available. This may account for the high recurrence rates and failed rehabilitation programs of some athletes with longstanding going pain.

### Current evidence for exercise therapy for groin pain in an athletic population

Currently, common strategies used in the management of athletic groin pain rely on composite therapies comprising pharmacotherapy, surgery, active and/or passive therapy [13-15]. Exercises prescribed as part of rehabilitation for groin pain are often derived from the personal experience of the treating therapist without a standardized or evidence-based rehabilitation protocol [16].

Until recently, there has been a scarcity of literature on effective treatment for athletes with groin pain. Two systematic reviews on athletic groin pain [13,17] have been published in 2008. The first by Choi and colleagues [17] reviewed the evidence for the treatment of osteitis pubis and osteomyelitis of the pubic synthesis in athletes and the second by Jansen and colleagues [13] reviewed the evidence on the treatment of longstanding groin pain in athletes.

Choi's [17] review included case studies and reported that strong recommendations could not be made due to low level study design. From this review the recommendation was made that the first line of therapy in the treatment of osteitis and osteomyelitis should consist of conservative therapy. However the authors did not give further details on how to apply this into clinical practice.

Jansen's [13] review acknowledged that the complexity of longstanding groin pain in athletes does have a direct effect on treatment. As such the authors summarised that treatment can involve both conservative and surgical interventions. Their review demonstrated that often conservative measures are initially trialled, frequently consisting of an initial rest period followed by physiotherapy. Commonly physiotherapy treatments aimed at improving the stability of the pelvis and hip are trialled before surgical interventions are considered. This strategy is commonly seen in clinical practice as has been shown by Pizzari and colleagues [18] in their qualitative study of prevention and management of osteitis pubis in the Australian Football League. Jansen's review [13] provides a valuable overview of exercise interventions for groin pain in athletes, but it does not examine the specific features of the exercise programs. There is a need for a systematic review that specifically aids the clinician in applying the best available evidence for exercise prescription for athletes presenting with groin pain.

The primary aim of this systematic review was to evaluate the available evidence on the effectiveness of exercise therapy for groin pain in athletes. The secondary aim of this review was to identify the key features of the exercise interventions which have been reported in the literature for the management of groin pain in athletes, to aid the clinician in making evidence-based decisions.

## Methods

### Search Strategy

A series of standard, reproducible, systematic review processes were undertaken for this literature review. As the first step in this process, the systematic review objectives were deconstructed into population, intervention, comparison groups and outcomes (PICO). The PICO format provides a framework for deconstructing review parameters into distinct categories. This is presented in Table 1.

**Table 1:** PICO Format

**P**opulation	Athletes complaining of groin pain
	*Athlete: an individual who regularly participates (i.e. trains and competes) in an athletic activity as part of their exercise routine*
	*Groin Pain: pain arising from the anterior groin region around the pubis and inguinal ligament, adductor region and lower abdominal musculature*
**I**ntervention	Exercise Therapy

**C**omparison	Other forms of conservative or surgical management

**O**utcomes	*Subjective *e.g. Pain scores *Objective *e.g. Adductor muscle length and strength tests *Functional outcome *e.g. Return to sports

Searches were performed from 1990 to February 2009 on the following databases: MEDLINE, CINAHL, PubMed, SPORTSDiscus, Embase, AMED, Ovid, PEDro, Cochrane Controlled Trials Register and Google Scholar. The reference lists of retrieved articles were reviewed, in order to identify relevant studies. Identified duplicates were removed to create a master list of identified articles. A wide range of key words were utilized to search relevant databases. These key words were grouped into three different categories to ensure that a broad search would be conducted. Category one represented key words within the category "groin" ("groin injury", "groin strain", "groin pain", "adductor muscle strain", "iliopsoas", "osteitis pubis" and "pubic bone stress injury"). Category two represented key words within the category of "exercise" ("physiotherapy", "physical therapy", "exercise" and "rehabilitation"). Category three represented key words within the category "population" ("athlete" or "sports player"). Truncation symbols were utilized as appropriate across different databases.

### Selection of studies

All studies published in the English language that used exercise therapy as an intervention to treat groin pain in athletes were included in this review. Both acute and long standing groin pain were included.

### Groin Pain Definition

Groin pain could be defined by any of the following; 1) subjectively by the athlete as pain arising from the areas of the iliopsoas and adductor muscle groups or the lower abdominal musculature, 2) objectively as pain on palpation of adductor or lower abdominal musculature, pubic synthesis or pubic bone, 3) positive adductor muscle length or strength tests or, 4) pain in the areas as above affecting an athlete's function or a component of their athletic activity.

Groin pain can encompass a wide range of pathologies and some of these may not respond favourably to exercise intervention. Therefore studies which included groin pain from other anatomical or pathological sources including any type of hernia, piriformis syndrome, sacroiliac dysfunction, nerve entrapments, referral from the lumbar spine, rectal or testicular referral and co-existing fractures of the pelvis or lower limb were excluded. Studies which included groin pain and were associated with any abnormality or pathology relating to the hip were also excluded.

### Hierarchy of evidence

As this review addressed an effectiveness question, only research studies from a quantitative research paradigm were included. The National Health and Medical Research Council (NHMRC) hierarchy of evidence was used to evaluate identified studies [19], (refer to Table 2). Preliminary searching found minimal level I and II evidence. Therefore, in order to address the aims of this review, all study designs from Level I to IV were included. The benefits of this approach are to gain insight in what literature to date has shown and thus aid in developing recommendations for further research.

**Table 2:** NHMRC Hierarchy of Evidence

**Level of evidence**	**Type of study design**
**I**	A systematic review of level II studies
**II**	A randomised controlled trial
**III-1**	A pseudorandomised controlled trial
	(i.e. Alternate location or some other method)
**III-2**	A comparative study with concurrent controls:
	▪ Non-randomised, experimental trial
	▪ Cohort study
	▪ Case-control study
	▪ Interrupted time series with a control group
**III-3**	A comparative study without concurrent controls:
	▪ Historical control study
	▪ Two or more single arm study
	▪ Interrupted time series without a parallel control group
**IV**	Case series with either post-test or pre-test/post-test outcomes

### Methodological Evaluation

The methodological evaluation of included studies was conducted by using a modified quantitative critical appraisal tool developed by Law et al [20]. This generic critical appraisal tool was modified to contain twelve criteria, each representing key elements of the methodological quality of a research study. Each criterion was given a score of one. Studies were appraised by two independent reviewers [ZM, SK] and provided with a quality score. Any disagreements were resolved through discussions until consensus was achieved. Due to a mixture of research designs, and the nature of the generic critical appraisal tool used, some criteria were not applicable to all study designs and hence the overall quality score varied between study designs. A copy of this tool is provided in Appendix 1, Additional file 1.

### Data Extraction

Data was extracted by two reviewers [ZM, LGP] in reference to the characteristics of exercise interventions and further divided into two main categorizes. The first encompasses the features of the exercise interventions including type and indication for progression of exercise, as well as intensity, frequency and duration of exercise. The second encompasses how the exercise intervention was delivered, including the nature of any supervision provided and additional co-interventions utilised. This approach enabled the secondary aim of this review to be addressed.

### Body of evidence framework

The National Health and Medical Research Council (NMHRC) in Australia have produced numerous resources to better understand research evidence and integration of research evidence into clinical practice. NHMRC is currently in the process of developing a "body of evidence matrix" [21]. The body of evidence matrix considers all evidence dimensions, of all included studies, and is a highly relevant tool for use in systematic reviews which utilise all available evidence. There are five key components which make up the body of evidence for each recommendation. The first component relates to the evidence base which can be assessed in terms of quantity of evidence, level of evidence and quality of evidence. The second component relates to whether the findings are consistent across the included studies, which may encompass a range of study populations and study designs. The third component relates to clinical impact, which is a measure of the possible benefits of applying these findings to a population. The fourth component relates to generalisability, which relates to the generalisability of the findings of the research (subjects and settings of included studies) to those of the recommendation. The fifth and final component relates to applicability to the Australian health care setting and this component was excluded to maintain an international perspective for the results of this review.

## Results

### Search Results

**Figure 1:**
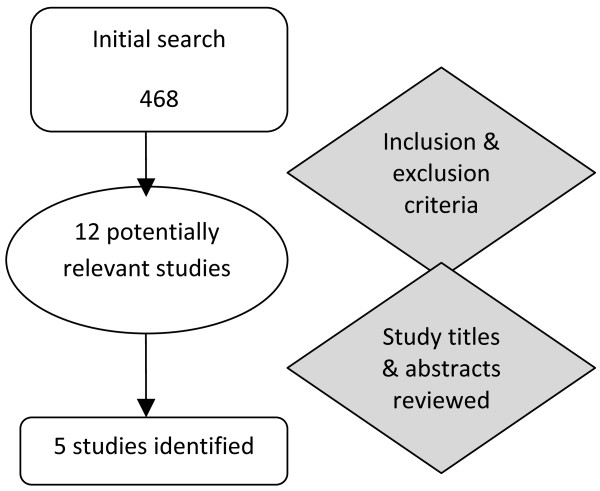
Search Results

The search strategy initially identified 468 studies, 12 potentially relevant. From reviewing the titles and abstracts, six of these 12 studies were excluded as they failed to provide adequate information on their intervention or reported incomplete results. One study was excluded because its population included athletes who were positive on hernia testing [22]. Overall five studies were identified as fulfilling all the inclusion criteria for this systematic review [14,15,23-25]. All five research studies were reported in prominent medical journals and were published from 1999 to 2007. Figure 1 provides an overview of the search results. Table 3 provides an overview of the characteristics of included studies. Only one randomised control trial [23] was identified in addition to four case series and/or studies [14,15,24,25].

**Table 3:** Characteristics of included studies

	**Holmich et al**. [23]	**McCarthy & Vicenzino **[24]	**Rodriguez et al**. [14]	**Wollin & Lovell **[25]	**Verrall et al**. [15]
**Aim**	*To compare an active training program with a conventional physiotherapy program in the treatment of severe & incapacitating adductor-related groin pain in athletes*	*To describe an alternate approach to assessment & treatment of osteitis pubis*	*To describe the pathomechanics, diagnostic procedures, classification & conservative management of osteitis pubis syndrome in elite soccer players*	*To report on successful rehabilitation outcomes and two new possible clinical indicators for return to football post osteitis pubis*	*To determine the outcome of treating chronic groin injury using a conservative (nonsurgical) treatment program*

**Study Design**	Randomised Clinical Trial	Case Report	Case Report	Case Series	Case Series

**Sample Size**	68	1	35 (over 8 year period)	4	27

**Location of Study**	Denmark	Australia	Mexico	Australia	Australia

**Sports Identified**	Soccer & other*	Gaelic football	Soccer	Soccer	Australian rules Football

*Other included running, tennis, European handball, badminton, ice hockey, basketball, horseriding, rugby

### Methodological quality of included studies

The quality scores derived from the critical appraisal are summarised in table 4. Scores were converted to percentages for ease of interpretation and to acknowledge different research study designs. Findings from this process revealed a wide variability in quality scores indicating variable methodological quality. Scores ranged from a low 40% [24,25] to a high 83% [23].

**Table 4:** Methodological Quality of included studies

	**Criteria**													**Scores (%)**
**Study**	*1*	*2*	*3*	*4*	*5*	*6*	*7*	*8*	*9*	*10*	*11*	*12*	**Score**	
Holmich et al. [23]	Y	Y	Y	Y	N	Y	N	Y	Y	Y	Y	Y	10/12	**83.33**
	
Verrall et al. [15]	Y	Y	Y	N	N	N	N	Y	Y	Y	Y	Y	8/12	**66.67**
	
Rodriguez et al. [14]	Y	Y	Y	NA	N	Y	N	N	N	NA	Y	Y	6/10	**60.00**
	
Wollin & Lovell [25]	N	Y	Y	NA	N	Y	N	N	N	NA	Y	Y	4/10	**40.00**
	
McCarthy & Vicenzino[24]	Y	N	N	NA	N	N	N	N	N	NA	Y	Y	4/10	**40.00**

NA = not applicable

### Primary aim: The overall effectiveness of exercise therapy for groin pain in athletes

All studies included in this review provided evidence that an exercise intervention can lead to favourable outcomes in terms of return to sport. In addition, two studies [14,15] identified that exercise therapy resulted in successful symptom remission in soccer and Australian football players respectively.

### Secondary aim: Identify the key features underpinning exercise programs used in the management of groin pain in athletes

Table 5 and 6 summarise data collected in reference to features of the exercise interventions.

**Table 5:** Delivery of exercise interventions

**Study**	**Delivery of exercise interventions**		
	
	**Nature of supervision**		**Co- intervention**
		
	**Supervised by**	**Number in group**	
Holmich et al. [23]	Physiotherapist	2–4	Jogging/cycling if pain free.

McCarthy & Vicenzino [24]	Physiotherapist	1	1) Massage and stretching of tight muscle groups.
			2) Jogging/running.

Rodriguez et al. [14]	NR	NR	1) Anti-inflammatory medication
			2) Electro-therapy
			3) Cycling or swimming
			4) Walking or jogging

Wollin & Lovell [25]	Physiotherapist	1	1)Massage
			2) Muscle energy techniques
			3) Manipulation
			4)Ultra-sound
			5) Neoprene shorts
			6) Running and cycling

Verrall et al. [15]	NR	NR	1) Swimming
			2) Upper body weights
			3) Cycling

NR = not recorded

**Table 6:** Features of exercise interventions

**Study**	**Features of exercise interventions**						
	
	**Intervention**			**I**	**F**	**Duration**	
				
	**Adequately described -could reproduce? Y/N**	**Summary**	**Method of progression**			**Time (mins)**	**Total weeks**
Holmich et al. [23]	Y	1) Strengthening hip abductors/adductors (isometric and isokinetic).	Standardised progression of difficulty and resistance after 2 weeks	A	3	90	8–12
		2) Abdominal strengthening.					
		3) Balance training.					

McCarthy & Vicenzino [24]	N	1) Repetition of kicking and running movement patterns.	Once functional control was obtained	NR	1.4	NR	5
		2) Strengthening gluteus medius & transverse abdominus (isometric and isokinetic).					
		3) Home muscle stretching program.					

Rodriguez et al. [14]	N	1) Stretching exercises	When pain free	NR	NR	NR	3.8–10
		2) Strengthening hip abductors/adductors (isokinetic).					
		3) Abdominal strengthening.					
		4) Various running drills.					

Wollin & Lovell [25]	N	1) Transverse abdominus muscle training.	1) When pain free	A	NR	NR	10–16
		2) Strengthening hip abductors/adductors, hip flexor and extensor muscles (isometric and isokinetic)	2) When bench-mark strength and function achieved				

Verrall et al. [15]	N	1) 12 week rest period from running and weight-bearing activities.	When pain free	A	NR	5*	12 weeks
		2) Core stability program commencing between 3 and 6 weeks.					
		3) 'Versa climber' stepping machine commencing at 6 weeks if pain free.					

I = Intensity A: more than 3 sets of 10 repetitions or more than one minute of continuous work)

B: less than 3 sets of 10 repetitions or less than one minute of continuous work)

F = Frequency (sessions per week)

* = Increased by 1 minute per day

NR = not recorded

#### Type of exercise and how it was progressed

Four of the five studies reviewed included a strengthening component as a part of their exercise intervention [14,23-25]. Strengthening exercises used in these four studies targeted one or more of the hip flexors, abductors or adductors and the deep or superficial abdominal musculature. All four of these studies used through-range (isokinetic) strengthening. Three out these four studies began with static (isometric) contractions and then progressed their athletes to through-range movements using functional, standing positions similar to those required by their sport [23-25].

Only one of the five studies used a predetermined graduated exercise protocol for their athlete [23]. The remaining four studies used one or more of the following criteria as an indication of the need for progression of exercise; 1) the absence of pain with exercise, 2) the acquisition of functional control and 3) the ability to complete a functional exercise or a set number of repetitions of an exercise.

#### Intensity, frequency and duration of exercise interventions

Only one study provided enough detail of the frequency and duration of their intervention to claim that their intervention was reproducible [23]. The results of this study suggest that 90 minutes of strengthening exercises for the hip and abdominal musculature performed three times per week for an overall duration of 8–12 weeks is sufficient to attain good outcomes in terms of return to sport without groin pain.

No study appropriately reported the intensity of their exercise interventions. Measures such as resistance, weight or perceived exertion were not recorded in any study. Only two studies recorded the frequency of sessions of exercise performed per week [23,24]. The overall duration of exercise interventions across all five studies ranged from 3.8 weeks to 16 weeks.

#### Delivery of exercise interventions

Two of the five studies did not report who supervised the exercise program [14,15]. Of the remaining three studies, all were supervised by a Physiotherapist. Group sizes were small, ranging from one to four in the studies that reported this information.

All five studies reported the use of one or more co-interventions. These co-interventions ranged from massage and manipulation to anti-inflammatory medication. Four out of the five studies included either jogging or running as a co-intervention [14,23-25]. In addition, four out of the five studies included cycling as a co-intervention [14,15,23,25]. Only two out of the five studies included passive treatment techniques such as massage and mobilisation as co-interventions [24,25]. Only one of the five studies used medication (anti-inflammatory) as a co-intervention [14].

### NHMRC body of evidence framework

The evidence identified from this systematic review has been presented in the NHMRC body of evidence framework in table 7. Based on these composite findings, derived from the four components of evidence, it is recommended that exercise therapy for groin injury of both acute and chronic nature, could be considered as a viable management option for groin pain in athletes. It is recommended that due to limitations within the existing evidence base, regular review of progress and evaluation of outcomes be undertaken as part of implementing this recommendation.

**Table 7:** NHMRC body of evidence framework

**Component**	**Grade**	**Comments**
**Evidence Base**	**D-Poor**	• Total of 5 studies
	*Level IV studies or level I to III studies with risk of bias*	• Total of 135 male athletes
		• L II: 1 study
		• L IV: 4 studies
		• Quality of studies (table 4 in text)

**Consistency**	**B-Good**	• Population: Australian football, soccer & others
	*Most studies consistent and inconsistencies may be explained*	• Outcomes used: 4/5 studies used return to sport; 4/5 used subjective scores
		• While most studies consistently reported on the positive effects of exercises for groin pain, there is variability in the populations included, interventions provided and outcomes measured potentially leading to heterogeneity

**Clinical Impact**	**C – Satisfactory**	• Presently only one high level, high quality publications in the literature
	*Moderate*	• The clinical impact from available evidence base is only satisfactory as vital information on effect size, comparison with other management options are missing
		• The current evidence base also focused on mainly subjective measures of outcomes

**Generalisability**	**B-Good ** *Population(s) studied in the body of evidence are similar to the target population*	• Population studied in the evidence base is similar to the target population • However, the current evidence base lack clarity in terms of diagnosis, injury periods and predominately focused on the male sporting population

**Grade of Recommendation**	**D- Poor**	• Limited number of studies were identified from the literature
	*Body of evidence is weak and recommendation should be applied with caution*	• Overall, these studies were low level and were of moderate quality
		• While the findings were consistent, there were issues with varying diagnostic criteria, poor description of interventions, differing outcome measures and lack of long term follow up

I = Intensity A: more than 3 sets of 10 repetitions or more than one minute of continuous work) B: less than 3 sets of 10 repetitions or less than one minute of continuous work) F = Frequency (sessions per week) * = Increased by 1 minute per day NR = not recorded

## Discussion

The primary aim of this systematic review was to evaluate the overall effectiveness of exercise therapy for groin pain in athletes. Despite the scarcity of controlled trials in this area of research, this review helps to affirm commonly held clinical opinions that exercise therapy can play a crucial role in attaining positive outcomes for athletes with groin injury.

All studies included in this review provided some evidence that an exercise intervention can lead to favourable outcomes, in terms of return to sport. Despite favourable short-term results, only two studies provided support of ongoing positive benefits [15,25]. Wollin and colleagues [25] reported that at one year post intervention no athlete had subjectively reported re-aggravation of symptoms. Verrall and colleagues [15] reported a 100% return to sport rate at two years post intervention but this may not be surprising considering the sample population were professionally paid athletes, with a vested interest in returning to sport.

Due to the diverse nature of the primary research evidence, there was a great deal of variability in terms of interventions and co-interventions reported. Even though all five studies utilized differing exercise interventions with different features, one consistent finding across all the studies was the need for progression throughout the intervention period. This is clinically significant as progressions take into account gains attained throughout the treatment period and may add to the external generalisability of the findings of this review.

The secondary aim of this review was to identify the key features of the exercise interventions which have been reported in the literature in the management of groin pain in athletes. Despite the variability in interventions used in the studies and the inconsistent reporting of the components of the exercise programs, some key findings could be reported in reference to the features of the exercise interventions used across all five studies.

### Type and progression of exercise

The available evidence suggests that strengthening exercise of some type is an important component of an effective exercise intervention for groin pain. The variability between studies in terms of the muscle groups focused on and the chosen strengthening dose (intensity, repetition, frequency and duration) makes it difficult to conclude on a specific muscle group to focus on, or a specific strengthening dosage. However many clinicians would be interested in the finding that of the four studies that utilized strengthening exercises, all used through-range (isokinetic) exercises. Three of these studies began initially with static (isometric) contractions before progressing to functional standing positions. These exercises can be easily reproduced in the clinic and taught to athletes.

### Intensity, frequency and duration of exercise

Considering no study reported intensity and only two [14,23] reported frequency it is difficult to draw conclusions. Duration of exercise intervention is the only parameter which was reported consistently enough across all five studies to allow conclusions to be drawn. The variation in duration of exercise interventions used may reflect a variation in the severity of groin pain across the different sporting populations in each study and the inclusion of retrospective study designs. This could have an impact on the time needed for resolution of symptoms and hence intervention period. Overall three [14,24,25] of the five studies included in this review were retrospective in design and this, along with the inclusion of multiple grades of groin pain may explain the large variation in duration of interventions across the studies.

### Method of delivery of exercise

The available evidence suggests that exercise interventions are most effective when administered in small groups of one and four athletes, and applied in combination with established co-interventions. The use of jogging, running or cycling as a co-intervention is a common feature of the five studies, all of which reported positive outcomes regarding return to sport. There is less evidence to support the use of passive treatment and medication as co-interventions to an exercise program for groin pain. These findings will be of interest to clinicians who wish to make an evidence-based decision regarding the use of co-interventions.

Of the studies reporting who supervised their exercise programs, all were supervised by Physiotherapists. This is important information in terms of service delivery. Although the research suggests that exercise for groin pain is effective, the supervision of the exercise and co-interventions by an expert in exercise prescription such as a Physiotherapist may be important.

### Limitations

As with any systematic review, this review has its own limitations. There is a profound lack of high level, high quality primary evidence to support exercise therapy for groin pain in athletes. Systematic searching of the literature identified only one level II study. Furthermore the methodological quality of the evidence base is only moderate. Additionally, there are issues with variations in diagnostic criteria between studies, lack of adequate recognition and reporting of pre-existing injuries, small sample sizes and lack of true control groups. The lack of detail in the reported interventions in most studies makes replication in clinical practice difficult. The lack of utilisation of psychometrically sound outcome measures and absence of long term follow up is also of concern. While these limitations provide drivers for future research which can aim to overcome these flaws, the current available evidence, limited at it may be, provides useful information to help guide practice. The use of NHMRC body of evidence framework provides an ideal mechanism for embracing multiple dimensions of evidence, rather than the traditional model of basing recommendation on the hierarchy and quality of evidence.

## Conclusion

### Implications for clinical practice

Best available evidence to date, with its limitations, continues to support common clinical practice of exercise therapy as a key component of rehabilitation for groin pain in athletes. Overall, the available evidence suggests that exercise, particularly strengthening exercise of the hip and abdominal musculature could be an effective intervention for athletes with groin pain. The evidence suggests that this strengthening exercise may need to be progressed (from static contractions to functional positions) and performed through range. There is currently no clear evidence regarding the most effective intensity and frequency of exercise, because of a lack of reporting in the primary literature. The evidence suggests that a duration of intervention of between 3.8 to 16 weeks may be required for exercise intervention to be effective. This is in stark contrast to routine practice, where there is often great pressure on all concerned to return an athlete to their sport. This, along with the financial pressure of professional sport can lead to shortened periods of intervention.

Interpretation and implementation of these findings into clinical practice will rely on the clinical reasoning and expertise of individual clinicians, who need to tailor these findings to suit the individual requirements of athletes. This process builds on the philosophy of Evidence Based Practice.

### Implications for research

The current evidence base for exercise therapy for groin pain is profoundly limited and relies greatly on clinical expertise and experiential knowledge. More randomised controlled trials, with clearly defined diagnostic groups and detailed exercise features, are required in order to inform rigorous and precise recommendations for exercise prescription for groin pain in athletes.

## Competing interests

The authors declare that they have no competing interests.

## Authors' contributions

ZM conceptualized the topic and devised the search strategy and carried out the initial search. SK, together with ZM assessed inclusion of studies into review and independently assessed quality of studies. Data extraction for the characteristics of exercise intervention was done by LGP together with ZM. ZM and SK contributed equally to initially draft, LPG together with ZM and SK were equally involved in the revision of manuscript as per reviewers' comments.

## Supplementary Material

Additional File 1 **Modified Critical review form (Law et al 1998) – Quantitative studies.** Click here for file
